# A New Era in the Management of Hypertrophic Cardiomyopathy

**DOI:** 10.31083/RCM44503

**Published:** 2025-10-30

**Authors:** Ana Perez-Asensio, Helena Llamas-Gomez, Ana Lara-Palomo, Alberto Villar-Ruiz, Cesar Jimenez-Mendez, Julian Palomino-Doza

**Affiliations:** ^1^Cardiology Department, Hospital Universitario Puerta del Mar, 11007 Cadiz, Spain; ^2^Cardiology Department, Hospital Universitario Virgen del Rocío, 41013 Sevilla, Spain; ^3^Cardiology Department, Hospital Universitario Doce de Octubre, 28041 Madrid, Spain; ^4^Radcliffe Department of Medicine, Division of Cardiovascular Medicine, University of Oxford, OX1 2JD Oxford, UK

**Keywords:** hypertrophic cardiomyopathy, sudden cardiac death risk stratification, septal reduction therapies, myosin inhibitors, gene therapy

## Abstract

Hypertrophic cardiomyopathy (HCM) is a prevalent cardiac disease characterized by marked phenotypic variability. Recent advances in diagnosis and treatment have allowed a personalized approach to the treatment of this disease. Depending on the predominant phenotype, management can be tailored to address left ventricular outflow tract obstruction, heart failure, arrhythmia control, and/or sudden cardiac death prevention. This review highlights recent advances that have transformed the therapeutic landscape of HCM. Modern imaging techniques have improved sudden cardiac death risk stratification. The development of myosin inhibitors represents a paradigm shift in the treatment of symptomatic obstructive HCM. Invasive septal reduction techniques have also evolved, with novel approaches such as percutaneous intramyocardial septal radiofrequency ablation and transapical beating-heart septal myectomy. Finally, gene-targeted therapies including replacement, editing and silencing approaches, are emerging as promising strategies for HCM management.

## 1. Introduction 

Hypertrophic cardiomyopathy (HCM) constitutes the most frequently inherited cardiomyopathy with an estimated adult prevalence of 0.2% [1]. HCM in adults is defined by a left ventricular (LV) wall thickness ≥15 mm in any myocardial segment that is not solely explained by abnormal loading conditions. With lesser degrees of wall thickening (13–14 mm), other variables, such as family history, genetic findings and ECG abnormalities, must be evaluated. In children, the diagnosis of HCM requires an LV wall thickness more than 2 standard deviations above the predicted mean (z-score >2) [1]. It presents a highly variable clinical course, ranging from a completely asymptomatic and stable condition, to one with severe outcomes [2]. Phenotype spectrum includes, amongst many other manifestations, left ventricular outflow tract obstruction (LVOTO), heart failure and arrhythmias, which may lead to sudden cardiac death (SCD). A tailored management approach results in improved morbidity and mortality outcomes [3].

Since the first descriptions of obstructive HCM (oHCM) and non-obstructive HCM (nHCM), there has been a revolution in the understanding of its pathophysiology [4, 5]. This has led to substantial changes in diagnosis, treatment, and SCD risk assessment [1]. Advances in imaging techniques, especially cardiac magnetic resonance (CMR), have provided key insights [6]. New risk assessment tools and algorithms have been developed [7]. Myosin inhibitors (MI) have transformed the treatment algorithm of patients with oHCM; being the first pharmacological group to act upon HCM’s pathophysiological mechanism [8]. Many interventional alternatives to Morrow classical septostomy have emerged [9]. Finally, better understanding HCM’s underlying genetic mechanisms has allowed the development of gene therapy (GT), raising the question of whether some HCM patients may be definitively cured in a near future [10].

All of the previous advances, has increased the need for clinicians to be fully updated to offer the best personalized integral management to HCM patients.

## 2. Sudden Cardiac Death Risk Stratification 

Annual SCD in HCM is estimated to be around 0.5–0.8% in adults and 1.2–1.5% in children. It may occur as the initial presentation of HCM [2, 11], being ventricular tachycardia/fibrillation (VT/VF) the most common causes of SCD. Implantable cardioverter-defibrillators (ICD) are an effective therapy for managing life-threatening ventricular arrhythmias [12]. Nonetheless, risk stratification is still challenging and controversial [13].

**Fig. 1.**
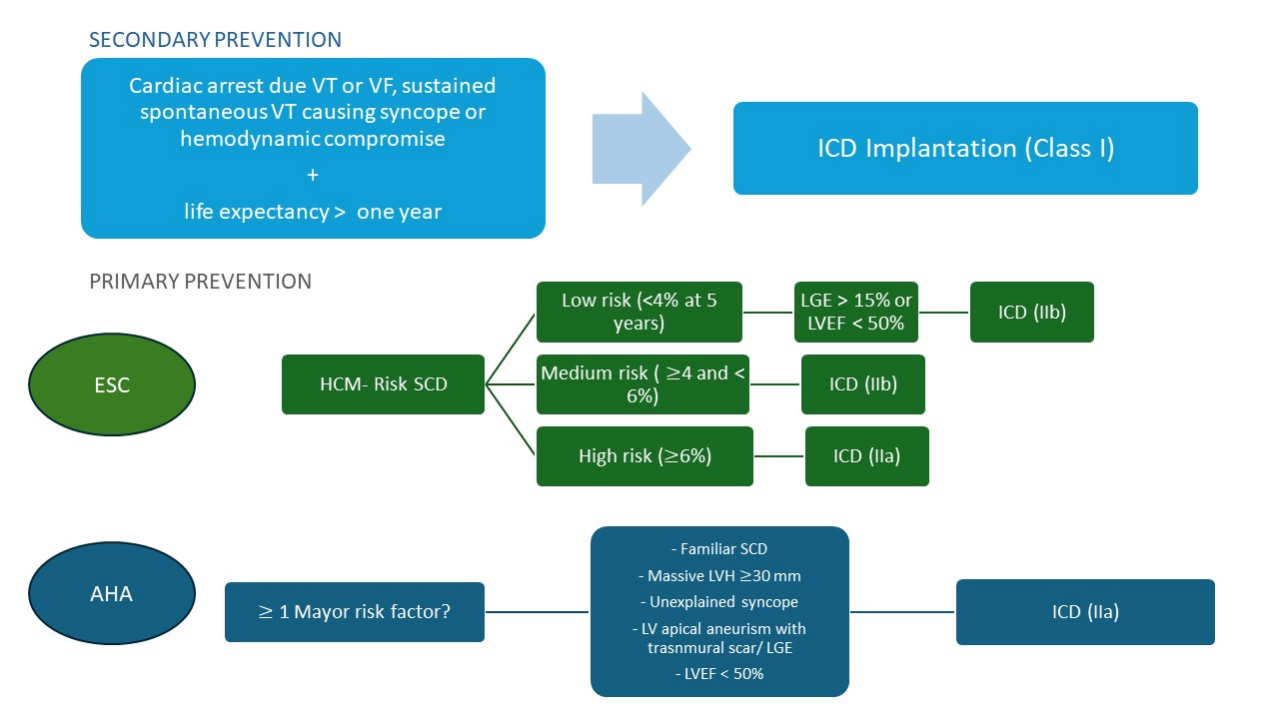
**Flowchart of sudden cardiac death risk assessment and implantable cardioverter-defibrillator implantation indications according to European (2023) and American (2024) guidelines**. AHA, American Heart Association guidelines; ESC, European Society of Cardiology guidelines; HCM, Hypertrophic Cardiomyopathy; ICD, Implantable Cardioverter-Defibrillator; LGE, Late Gadolinium Enhancement; LV, Left Ventricular; LVEF, Left Ventricular Ejection Fraction; LVH, Left Ventricular Hypertrophy; SCD, Sudden Cardiac Death; VF, Ventricular Fibrillation; VT, Ventricular Tachycardia.

Both ESC European Society of Cardiology guidelines (ESC) and American Heart Association guidelines (AHA) guidelines [1, 14] uniformly recommend, with class I indication, ICD implantation in secondary prevention. However, they diverge in their recommendations for primary prevention (Fig. 1). ESC guidelines endorse the use of the HCM Risk-SCD model for adults and the HCM Risk-Kids tool for patients under 16 to estimate 5-year SCD risk [7, 15]. ICD implantation is based on defined risk thresholds (low risk <4%, intermediate risk 4–6%, high risk ≥6%) [1]. In contrast, AHA guidelines adopt a major risk marker approach, using individual clinical features as a basis for shared decision-making [14].

Key differences exist between guidelines regarding the role of CMR, apical aneurysm [16, 17], and left ventricular ejection fraction (LVEF) [18, 19] (Table 1, Ref. [1, 14]). While the ESC guidelines view the previous as risk modifiers, emphasizing the limitations of current evidence and the need for individualized clinical interpretation, AHA guidelines consider them as independent risk factors that warrant ICD implantation. These differing strategies result in varying ICD implantation rates. The AHA method offers a higher sensitivity (≈95%) but lower specificity (≈78%), leading to more implants in low-risk patients. The ESC model favors specificity (≈92%), potentially missing some at-risk individuals [7]. Despite these contrasts, both approaches show similar effectiveness, with approximately six to seven patients needing ICD implantation to prevent one episode of VT/VF.

**Table 1.** **Comparison of key clinical risk factors for sudden cardiac death in hypertrophic cardiomyopathy from the ESC and AHA guidelines**.

Risk factor	ESC Guidelines (2023) [1]	AHA Guidelines (2024) [14]
Family history of SCD	SCD in ≥1 first-degree relative <40 years or at any age with confirmed HCM	SCD in ≥1 first-degree or close relative ≤50 years; multiple tertiary relatives also relevant
	Not independently associated with prognosis in pediatric HCM	
Unexplained syncope	Recent (<6 months)	Events >5 years ago less relevant
Age	Younger patients (≤15 years) at higher risk	Not explicitly discussed
	Age affects marker sensitivity	
Maximum LV wall thickness	Highest risk if ≥30 mm	≥30 mm major risk factor
		≥28 mm considered at physician discretion
		Pediatric: z-score ≥20 (or >10 + additional risk factors)
Left atrial diameter	Larger size linked to increased SCD risk	Not mentioned
LV apical aneurysm	Not listed	Regardless of aneurysm size
LVEF	Not emphasized	LVEF <50%
LVOTO	Uncertain impact of provocable LVOTO or treatment	Not mentioned
	Conflicting pediatric data	
NSVT	Independent risk factor	Higher risk if frequent (≥3 runs), longer (≥10 beats), faster (≥200 bpm); pediatric: >20% above baseline HR
	Exercise-related NSVT may increase risk	
	Frequency/duration not clearly predictive	
LGE	>15% LGE shared decision making	Extensive LGE

HCM, Hypertrophic Cardiomyopathy; LGE, Late gadolinium enhancement; LV, Left Ventricle/Left Ventricular; LVEF, Left ventricular ejection fraction; LVOTO, Left Ventricular Outflow Tract Obstruction; NSVT, Non-Sustained Ventricular Tachycardia; SCD, Sudden Cardiac Death.

### Future Directions in Sudden Cardiac Death Risk Stratification

Previous ESC and AHA SCD risk stratification models rely on regression analysis methods. However, emerging data derived from CMR, as well as genetic information, are becoming increasingly relevant for identifying arrhythmogenic risk. Looking ahead, new risk stratification paradigms that integrate old and new risk markers are needed. Machine learning and artificial intelligence (AI) offer the potential to synthesize complex data, delivering a more personalized risk assessment, particularly in patients with intermediate or uncertain risk profiles under conventional models.

#### Cardiovascular Magnetic Resonance 

CMR enhances SCD risk prediction in HCM by providing detailed myocardial tissue characterization. Parametric mapping techniques like late gadolinium enhancement (LGE), T1/T2 mapping, and extracellular volume (ECV) to quantify fibrosis, edema, and extracellular expansion linked to arrhythmic risk can be used [6]. An LGE affecting ≥15% of LV mass significantly increases the 5-year SCD risk, even among patients initially classified as low risk [20]. Additionally, the combination of LGE with elevated T2 mapping (≥44.9 ms) is associated with worse outcomes [21]. ECV correlates more strongly with sudden cardiac death risk than LGE alone [22]. Finally, native T1 mapping independently predicts adverse cardiovascular events more accurately than guideline-based risk scores [23]. The association between SCD risk and LGE in children is not well defined [14].

Taking into account these findings, both ESC/AHA guidelines support the use of CMR for SCD risk stratification in low to intermediate risk HCM patients [1, 14]. However, several uncertainties temper the integration of CMR clinical practice [24]. Quantification of LGE varies widely between centres, scans and software programmes, lacking standardization. Moreover, availability of CMR in low resource settings is limited. Before generalization of the recommendations, a consensus on how to incorporate these variables into decision algorithms is required.

#### Genotype Status

Genetic testing may provide prognostic insight into HCM. Carriers of pathogenic or likely pathogenic sarcomere gene variants generally face higher risks of adverse outcomes. Certain mutations and regions in genes such as *MYBPC3*, *TNNT2* and *MYH7* have been linked to a worse prognosis and increased arrhythmic risk [25, 26, 27].

Despite the previous guidelines, the most recent 2023 ESC guidelines have removed the presence of a single sarcomeric pathogenic variant as a standalone indication for ICD implantation in patients with intermediate SCD risk. Genetic findings are supportive but not determinative of eligibility [1]. This is due to association inconsistency across all patient groups. Moreover, additional factors such as polygenetic variants and diastolic blood pressure influence disease expression [28], making it challenging to attribute SCD risk to a single variant or genomic region alone [29]. Before integration into clinical algorithms, more investigation is needed in order to correctly delineate genotype–phenotype correlations in HCM.

#### Artificial Intelligence

In the years ahead, AI is poised to become a new tool for SCD risk assessment. It has proven useful in HCM screening through ECG analysis, with high specificity and negative predictive value [30, 31]. Although a validated AI tool specifically designed for SCD risk assessment in HCM is not yet available, several promising models exist. These include AI algorithms that identify high-risk ECG patterns, integrating clinical, genetic and imaging data to predict outcomes [32, 33, 34]. Model generalizability is however hindered in HCM due to limited sample sizes, variable disease expression, and complex phenotypes.

## 3. Pharmacological Therapy

### 3.1 Myosin Inhibitors

**Fig. 2.**
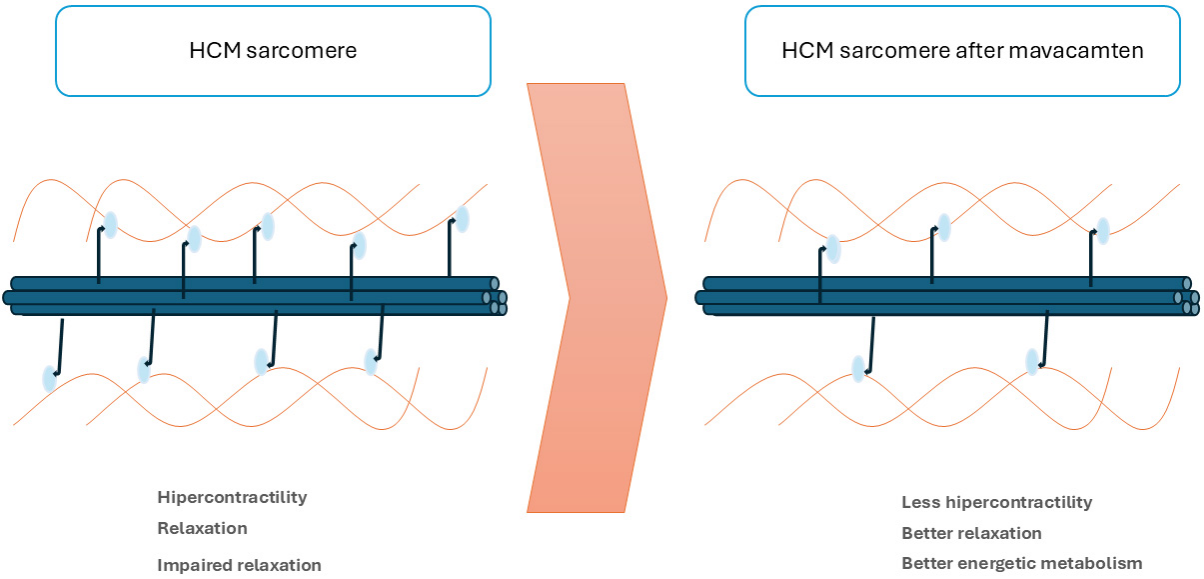
**Mechanism of action of myosin inhibitors**. HCM, Hypertrophic Cardiomyopathy.

MI are a new drug group, which selectively and reversibly inhibit cardiac myosin adenosine triphosphatase. They stabilize the super-relaxed state of myosin, thus reducing actin-myosin bridge formation and, consequently, the excessive myocardial contractility associated with HCM pathophysiology. This leads to a reduction in LVOTO and LV filling pressures. It also reduces myocardial energy demands and diastolic dysfunction [8, 35] (Fig. 2).

To date, there are two MI: mavacamten and aficamten. The main differences between them are shown in Table 2. Aficamten, a second-in-class allosteric MI was developed to improve the pharmacokinetic and pharmacodynamic properties of mavacamten. Their most frequent adverse reactions are dizziness (17%), dyspnea (12%), left ventricular systolic dysfunction (5%) and syncope (5%) [1, 35].

**Table 2.** **Main differences between myosin inhibitors**.

	Mavacamten	Aficamten
Half-life (days)	6–23	2.8
Drug interactions	*CYP450*	None
Dosage titration	Slow (4 weeks)	Quick (2 weeks)
Risk of LVEF <50%	+++	+
Efficacy in oHCM	↑ ↑	↑ ↑

LVEF, Left Ventricular Ejection Fraction; oHCM, Obstructive Hypertrophic Cardiomyopathy. +++: higher risk; +: risk but less than with mavacamten. ↑↑ higher efficacy.

#### 3.1.1 Myosin Inhibitors in Obstructive Hypertrophic Cardiomyopathy

Mavacamten and aficamten have demonstrated efficacy in oHCM across several trials (Table 3, Ref. [36, 37, 38, 39, 40, 41]). Mavacamten received FDA approval in 2022, while aficamten received approval in 2024. When published, the ESC guidelines were unable to recommend the use of MI as first-line medical therapy, due to the absence of direct head-to-head comparisons with other treatments available at the time. However, they did consider the evidence sufficiently robust to support their use as second-line therapy when optimal medical therapy with beta-blockers, calcium antagonists, and/or disopyramide was ineffective, poorly tolerated or contraindicated [1]. MI may be co-administered with beta-blockers or calcium antagonists. However, safety with negative ionotropic drugs (such as disopyramide) has not been established.

**Table 3.** **Main studies of Myosin Inhibitors in adults with obstructive hypertrophic cardiomyopathy**.

Study title	EXPLORER-HCM [36]	VALOR-HCM [37]	MAVA-LTE [38]	SEQUOIA-HCM [41]	FOREST-HCM [39]	MAPLE-HCM [40]
Drug	Mavacamten	Mavacamten	Mavacamten	Aficamten	Aficamten	Aficamten
Design	Double-blind Randomized	Double-blind	Open-label	Double-blind	Open-label	Double-blind
		Randomized	Extension	Randomized	Extension	Randomized vs metoprolol
		Placebo controlled, Cross-over at week 16		Placebo controlled		
N	251	112	231	282	213	175
Duration (weeks)	30	56	260	24	Ongoing. 48 week analysis.	24. *Final results pending publication.*
NYHA class	II–III	III–IV	II–III	II–III	II–III	II–III
Primary endpoint	pVO_2_ increase ≥1.5 mL/kg/min and at least one NYHA class	Proportion of patients undergoing SRT or remaining guideline elegible (↓)	Safety (☺)	pVO_2_	Safety (☺)	pVO_2_ (↑)
Secondary endpoints	LVOT gradient (↓)	LVOT gradient (↓)	LVOT gradient (↓)	LVOT gradient (↓)	LVOT gradient (↓)	LVOT gradient
	KCCQ-CSS (↑)	NYHA (↓)	NT-proBNP (↓)	NYHA (↓)	NYHA (↓)	NYHA
	NT-proBNP (↓)		NYHA (↓)	KCCQ-CCS (↑)	NT-proBNP (↓)	NT-proBNP
	hs-cTnI (↓)				hs-cTnI (↓)	KCCQ-CCS
					KCCQ-CCS (↑)	LV mass, LAVI

CPET, Cardiopulmonary Exercise Test; CV, Cardiovascular; cTnI, High-Sensitivity Cardiac Troponin I; EF, Ejection Fraction; KCCQ-CSS, Kansas City Cardiomyopathy Questionnaire–Clinical Summary Score; LVOT, Left Ventricular Outflow Tract; LVOT-G, Left Ventricular Outflow Tract Gradient; N, Patient Number; NRS, Numerical Rating Scale; NYHA, New York Heart Association; NT-proBNP, N-Terminal Pro-B-Type Natriuretic Peptide; pVO_2_, Maximum Oxygen Consumption; SRT, Septal Reduction Therapy; HCM, Hypertrophic Cardiomyopathy. ☺: Positive Clinical Trial Results. ↑: Increased ↓: Decreased.

In the EXPLORER-HCM trial, mavacamten improved functional capacity, symptoms, and quality of life. 37% of patients on treatment reached the primary endpoint, compared to 17% on placebo [36]. In VALOR-HCM, mavacamten showed a 77% reduction in the need for septal reduction therapy (SRT). In addition, improvements in functional class, LVOT gradients, and quality of life were observed [37]. Similarly, the SEQUOIA-HCM trial of aficamten reported increased peak VO_2_, significant reductions in LVOT gradients, better New York Heart Association (NYHA) class and improvement in symptom scores. The time that patients remained eligible for SRT shortened by 78 days [37].

Regarding long-term data, MAVA-LTE [38] is the five-year long-term study of mavacamten and FOREST-HCM [39] was the extension trial of aficamten. Both trials assessed long-term safety and tolerability, and included individuals who enrolled in previous pivotal studies. No treatment-related serious adverse events were identified. In MAVA-LTE, mean LVEF decreased by 11% from baseline to week 180, but remained within the normal range. In both studies, sustained reductions in LVOT gradient, and improvements in functional and hemodynamic parameters were observed during follow up. More real-world data from the Risk Evaluation and Mitigation Strategy (REMS) program support the safety and effectiveness of mavacamten. In 70% of patients, LVOT gradient reductions to <30 mmHg were observed. Most patients achieved benefits at low doses (5–10 mg) and only a low proportion of individuals had potential drug-drug interactions [42]. 4.6% of patients experienced reduced LVEF and 1.3% required hospitalization for heart failure [43].

Although not currently approved for use in children, pediatric studies with MI are underway. SCOUT-HCM [43] and CEDAR-HCM [44] are two currently recruiting clinical trials designed to evaluate the efficacy, safety, and pharmacokinetics of mavacamten in adolescents with symptomatic oHCM. Endpoints include, amongst others, changes in LVOT gradient, NYHA functional class and cardiac biomarkers. CEDAR-HCM will recruit children aged 6 to 11 years in its open-label extension study.

MAPLE-HCM is the first head-to-head trial comparing MI with beta-blockers. Preliminary results demonstrate that aficamten significantly improved peak VO_2_, with a favorable safety profile [40]. It remains to be determined if evidence will be sufficient to propose MI use as first-line monotherapy in oHCM.

#### 3.1.2 Myosin Inhibitors in Non-Obstructive Hypertrophic Cardiomyopathy

A shared underlying abnormality, regardless of hemodynamic features, in both oHCM and nHCM, has led to the clinical trials designed to assess MI efficacy in patients with nHCM (Table 4, Ref. [45, 46, 47]).

**Table 4.** **Main studies of Myosin Inhibitors in non-obstructive hypertrophic cardiomyopathy**.

	MAVERICK-HCM [45]	ODYSSEY-HCM [46]	ACACIA-HCM [47]
Drug	Mavacamten	Mavacamten	Aficamten
Design	Double-blind	Double-blind	Double-blind
	Randomized	Randomized	Randomized
	Placebo-controlled	Placebo controlled	Placebo-controlled
N	59	580	420
Duration (weeks)	16	48. Final results pending publication.	Ongoing. 72. Results expected in 2026
NYHA class	II–III	II–III	II–III
Primary endpoint	Safety (☺)	KCCQ-CSS (-)	KCCQ-CSS
		pVO_2_ (-)	
Secondary endpoints	pVO_2_ + NYHA (=)	VE/VCO_2_	pVO_2_, Ve/VCO_2_
	NT-proBNP and hs-cTnI (↓)	NYHA	NYHA
		NT-proBNP	LAVI
		KCCQ-CSS	NT-proBNP
			MACE

Hs-cTnI, High-Sensitivity Cardiac Troponin I; KCCQ-CSS, Kansas City Cardiomyopathy Questionnaire–Clinical Summary Score; LAVI, Left Atrial Volume Index; MACE, Major Adverse Cardiovascular Event; pVO_2_, Maximum Oxygen Consumption; VE/VCO_2_, Ventilatory Equivalent for Carbon Dioxide. ☺: Positive Clinical Trial Results; -: Negative Clinical Trial Results; =: indicates no significant change. ↓: Decreased.

Phase 2, MAVERICK-HCM [45] included 59 patients with symptomatic nHCM. Its primary outcome was safety. Mavacantem was well tolerated, with no differences in reported serious adverse events. Drug discontinuation because of LVEF reduction to <45% was uncommon (8%). Regarding efficacy, no difference between groups was observed in the composite functional outcome of maximum oxygen consumption (pVO_2_) + NYHA improvement. Exploratory analyses demonstrated reductions in N-terminal pro-B-type natriuretic peptide (NT-proBNP) and high-sensitivity troponin I levels, suggesting improvement in myocardial wall stress.

However, phase 3 ODYSSEY-HCM refuted MI clinical utility in nHCM [46]. Including 580 patients with nHCM in NYHA functional class II–III, it did not meet its primary outcome. No significant improvement was observed in Kansas City Cardiomyopathy Questionnaire–Clinical Summary Score (KCCQ-CSS) or in pVO_2_ after 48 weeks of treatment. Full results are still to be published. ACACIA-HCM [47] is an ongoing nHCM clinical trial assessing aficamten. It will include 420 patients and its primary endpoint is a change in KCCQ-CSS from baseline to week 36. Secondary endpoints are changes in maximal pVO_2_ and submaximal (Ve/VCO_2_) exercise capacity, proportion of patients with ≥1 class improvement in NYHA class, changes in left atrial volume index (LAVI), changes in NT-proBNP, and time to first major adverse cardiovascular event (MACE). The results are expected in 2026.

#### 3.1.3 Myosin Inhibitors in Structural Remodeling of Hypertrophic Cardiomyopathy

Small sub-studies using both echocardiography and CMR suggest that MI can promote favorable myocardial remodeling. Treatment has been associated with regression of septal wall thickness and indexed LV mass, reduction in left atrial volume, and improvement in diastolic filling patterns. Modest regression of fibrosis has also been observed through LGE and T1 mapping. Some patients showed normalization of previously abnormal ECGs [41, 48].

In the EXPLORERHCM CMR sub-study, after 30 weeks of mavacamten, the mean left ventricular mass index (LVMI) was decreased by 17.4 g/m^2^. The LAVI was also decreased by 10.3 mL/m^2^. There were no significant differences in LGE or ECV between groups at follow-up [48]. Similarly, in the SEQUOIA-HCM CMR sub-study, patients experienced reductions in LVMI (–15 g/m^2^) and LAVI (–13 mL/m^2^). Maximal wall thickness (–2.1 mm) and indexed extracellular volume mass (–3.9 g/m^2^) also decreased. Replacement fibrosis, assessed by LGE, remained stable with no statistically significant differences [41]. These findings may indicate that MI do more than just relieve symptoms. They may reverse key structural features of HCM phenotype, especially if treatment is initiated at earlier stages.

### 3.2 Other Emerging Pharmacological Therapies for Non-Obstructive Hypertrophic Cardiomyopathy

Negative results on the use of MI in nHCM may reflect fundamental pathophysiological differences between both HCM phenotypes. While obstruction drives symptoms in oHCM, diastolic dysfunction, caused by impaired myocardial bioenergetics, appears to be the main issue in nHCM. New treatment strategies specifically targeting nHCM are therefore needed.

IMPROVE-HCM evaluated ninerafaxstat, a cardiac myotrope in 67 patients with nHCM. The trial’s primary endpoint was safety and tolerability over 6 weeks. Secondary efficacy outcomes included ventilatory efficiency (VE/VCO_2_) and quality of life (KCCQ-CSS). Although results were promising, a phase 3 trial is yet to be initiated [49].

Potential use of sodium-glucose co-transporter 2 inhibitors (SGLT2i) in nHCM has also been evaluated. A clinical trial tested SGLT2i in patients with diabetes and symptomatic nHCM with preserved ejection fraction (EF). Its primary composite endpoint (a ≥1.5 improvement in E/e’ ratio and a reduction of at least one NYHA functional class after 6 months) was achieved by 70.8% of treated patients versus 4.2% of controls. Improvements were also noted in the 6-minute walk test and NT-proBNP levels. Recent real-world data further suggest potential benefits in reducing all-cause mortality and heart failure exacerbations [50, 51]. Confirmatory clinical trials are however still awaited.

## 4. Emerging Percutaneous and Surgical Techniques 

The 2023 ESC guidelines recommend SRT for patients with oHCM who have severe symptoms and a LVOT gradient >50 mmHg despite optimal medical therapy. With a lower strength of recommendation, SRT may also be considered in mildly symptomatic patients with resting or provoked gradients >50 mmHg when additional features such as atrial fibrillation, systolic anterior motion of the mitral valve, or left atrial enlargement are present. Currently, there is no evidence to support the use of SRT in asymptomatic individuals, regardless of imaging findings [1].

**Fig. 3.**
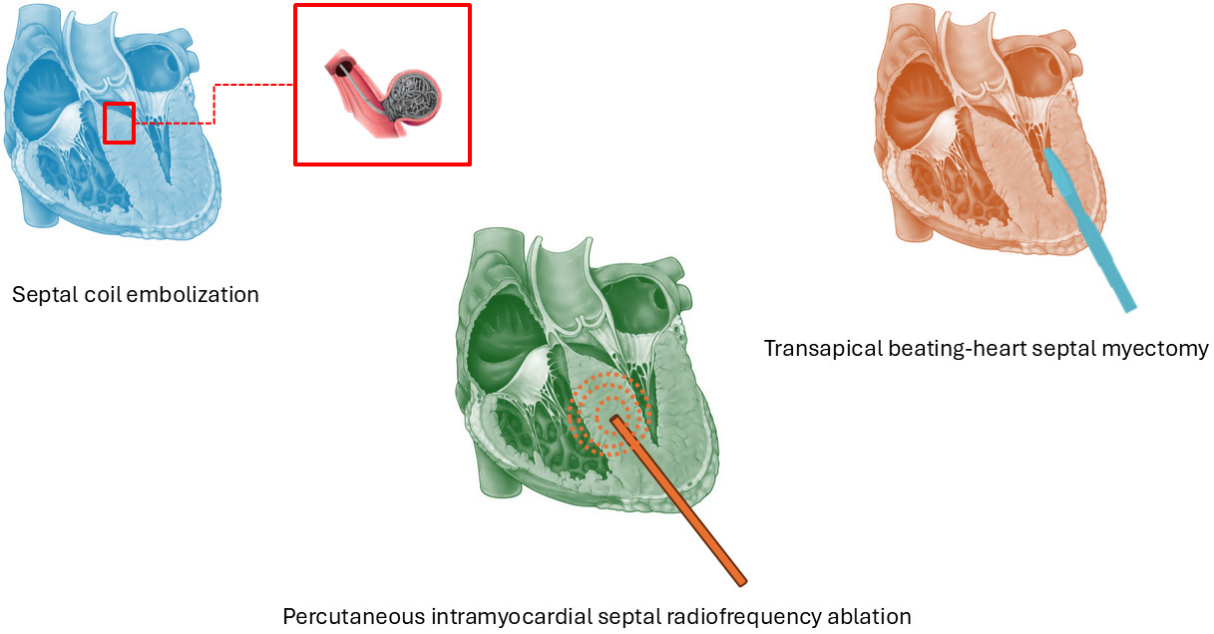
**Emerging percutaneous and surgical techniques**.

For decades, septal myectomy and alcohol septal ablation (ASA) have represented the two main options for STR. However, new strategies such as percutaneous intramyocardial septal radiofrequency ablation (PIMSRA), micro-coil septal embolization and transapical beating-heart septal myectomy (TA-BSM), have emerged as possible alternatives (Fig. 3). These techniques offer less invasive alternatives with promising efficacy. Compared to surgical myectomy, they present lower complication rates (5–10%), such as fewer conduction disturbances. They however tend to achieve a smaller reduction in LVOT gradient, especially in patients with complex anatomies [52]. Many of these new SRT techniques remain limited to specialized centres or are still in early-phase experience, with limited long-term data. Careful patient selection and individualized therapeutic planning remain essential. Moreover, MI and GT may promptly change LVOTO SRT indications.

### 4.1 Percutaneous Intramyocardial Septal Radiofrequency Ablation

PIMSRA is a catheter-based SRT that delivers controlled radiofrequency energy directly into the hypertrophied septum via a specialized needle-electrode system with expandable arms. This creates a customizable, elliptical-shaped lesion through localized myocardial heating (up to 80 °C), while sparing the endocardial surface and protecting the conduction system [53]. The procedure is performed via transapical access under echocardiographic and CT guidance. Studies have shown significant reductions in LVOT gradient and improvements in NYHA functional class [54]. In a large cohort of 200 patients, resting gradient decreased from 79.2 to 14.0 mmHg at 12 months, with no need for permanent pacemaker implantation [55]. Subsequent studies confirmed a gradient reduction of over 80% and notable symptom relief. When compared with surgical myectomy, PIMSRA offers similar efficacy with substantially lower complication rates [56, 57].

### 4.2 Septal Embolization With Micro-Coils

Septal embolization with micro-coils offers a mechanical alternative to ASA. It also imitates local myocardial infarction by selectively occluding a septal artery, but leaving behind the possible toxicity of alcohol [58]. It is less likely to affect the conduction tissue, thus reducing the risk of permanent atrioventricular block and the need for permanent pacing [58]. A 58% reduction in LVOT gradient has been previously reported.

### 4.3 Transapical Beating-Heart Septal Myectomy

TA-BSM is a novel surgical procedure that allows direct septal resection through mini-thoracotomy and LV apex access. It is performed while the heart is beating (without cardiopulmonary bypass), and employs a retractable coring device guided by real-time transesophageal echocardiography [59]. Recent studies report high procedural success (91%), significant LVOT gradient reduction, and low complication rates, including no need for permanent pacing [59, 60, 61]. TA-BSM appears effective even in complex anatomical variants and shows comparable short- and mid-term outcomes to conventional Morrow surgery, with the added benefits of less invasiveness and real-time control. Long-term data are still pending.

### 4.4 Other Emerging Approaches

Endocardial radiofrequency septal ablation offers precise endocardial targeting of the septal myocardium through guided electroanatomic mapping and intracardiac echocardiography. Short-term results have shown significant LVOT gradient reduction and symptomatic relief. However, comparative analyses suggest slightly lower efficacy than ASA and PIMSRA, in terms of septal thickness reduction [62, 63].

Robotic-assisted minimally invasive myectomy combines the precision of robotic platforms with reduced surgical trauma. Although still in the early stages of adoption, initial case series have demonstrated its feasibility and safety, particularly in elderly or high-risk patients [64].

High-intensity focused ultrasound employs extracorporeal energy delivery to induce precise myocardial necrosis. It has shown potential in canine models, presenting itself as an encouraging non-invasive alternative [65].

Transthoracic laser ablation (TTLA) involves percutaneous insertion of a laser fibre into the interventricular septum, delivering laser energy under magnetic resonance imaging or transesophageal echocardiogram guidance [66]. Its primary goal is to induce targeted myocardial coagulation necrosis, resulting in septal thinning and fibrosis. Preclinical animal studies have demonstrated feasibility and accuracy [67]. However, validation in humans is still required.

## 5. Gene Therapy

Multiple genes have been associated with HCM. According to the latest ClinGen Hereditary Cardiovascular Disorders Gene Curation Expert Panel, 29 genes have moderate, strong, or definitive evidence of association with HCM [68]. Most of these are sarcomeric genes, with *MYBPC3 *and *MYH7* accounting for approximately 70–80% of genotype-positive cases [26].

Genetic testing in HCM carries a Class I recommendation according to ESC guidelines. Approximately 30–40% of patients with HCM will have a pathogenic or likely pathogenic variant identified [1]. A positive result has significant clinical implications, especially for initiating cascade screening in family members. While genotype status is not yet fully integrated into treatment algorithms, the development of GT is expected to change this in the future.

GT aims to correct the disease at the DNA level [69]. It may be applied through different mechanisms: gene replacement, gene editing and gene silencing. While gene replacement and editing are typically designed as one-time therapies, gene silencing requires recurrent administration to maintain therapeutic effect. Adeno-associated virus (AAV) vectors are commonly used for cardiac GT delivery due to their natural cardiotropism.

∙ Gene replacement involves introducing a functional copy of a defective gene to enable the production of a normal protein [69]. It is mainly used for loss-of-function variants associated with haploinsufficiency. However, it may also serve as a disease-modifying strategy in dominant-negative or gain-of-function mutations by shifting the balance towards functional protein production [10].

∙ Gene editing aims to modify specific DNA sequences through insertion, deletion, or conversion of nucleotides [69]. The CRISPR-Cas9 system (Clustered Regularly Interspaced Short Palindromic Repeats-associated Cas9), guided by a single-guide RNA (sgRNA), introduces double-strand breaks at targeted genomic loci, enabling precise modifications [70]. Gain-of-function mutations can be disrupted by introducing frameshift-inducing insertions or deletions, while missense mutations may be corrected via targeted nucleotide substitutions. A critical challenge is to selectively target the mutant allele while preserving the wild-type allele [10].

∙ Gene silencing aims to prevent the production of defective protein while preserving normal protein expression [69]. It employs small interfering RNAs (siRNA) which bind complementarily to the mutated RNA selectively prompting its degradation. A critical challenge is to selectively target the mutant allele while preserving the wild-type allele [10]. This strategy is mainly employed in dominant-negative mutations. It requires the patient to be heterozygous with one functional allele that can tolerate haploinsufficiency.

### 5.1 Clinical Trials in Gene Therapy 

Numerous clinical trials employing different GT approaches have been conducted (Table 5).

**Table 5.** **Gene therapy strategies**.

Gene therapy strategy	Mechanism of action	Main use	Limitations	Preclinical evidence	Clinical translation
Gene Replacement 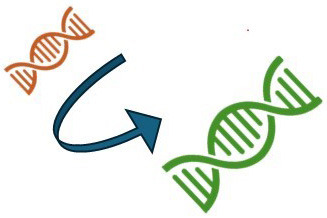	Delivery of a functional gene copy to restore normal protein expression.	Loss of function variants associated with haploinsufficiency.	- Dose dependent.	*MYBPC3* knock-in mice: ↑ mRNA and protein levels, no phenotype.	TN-201: AAV9-*MYBPC3*. Ongoing Phase 1 trial.
Gene Editing 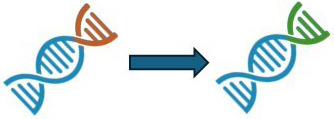	Precise correction of pathogenic variants by insertion, deletion or conversion of nucleotides.	Gain of function mutations and missense mutations.	- Allele specificity critical. - Atrial editing suboptimal. - Off-target editing.	*MYBPC3 p.W1098X:* correction of 3.65% of mutations; no phenotype. *MYH7 p.R403Q*: >70% correction, ↓hypertrophy.	Not yet in clinical trials.
Gene Silencing 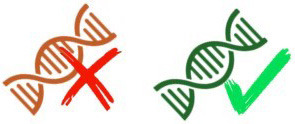	siRNA-mediated selective degradation of mutant mRNA while preserving wild-type expression.	Dominant-negative mutations.	Requires heterozygosity with tolerable haploinsufficiency. Recurrent administration for long term efficacy.	*MYH6 p.R403Q:* 80% mutant knockdown, no phenotype.	Not yet in clinical trials.

mRNA, Messenger Ribonucleic Acid; siRNA, Small Interfering Ribonucleic Acid. ↑: Increased ↓: Decreased.

Gene replacement strategies targeting *MYBPC3* have shown encouraging results in both animal and human models. Initial approaches used trans-splicing techniques, later evolving to full-length gene delivery. In a homozygous *MYBPC3* knock-in mouse model (*c.722G>A*), treatment with AAV9 carrying a therapeutic transgene led to increased messenger ribonucleic acid (mRNA) and protein expression, preventing the development of cardiac hypertrophy and dysfunction [71]. These findings were extended to human cardiomyocytes with truncating *MYBPC3* mutations [72]. Building on this progress, a recombinant AAV9 vector (TN-201) containing the *MYBPC3* gene is currently undergoing evaluation in a phase 1 clinical trial (NCT05836259) [73].

Gene editing approaches have also demonstrated therapeutic potential. One study targeting a premature stop codon mutation (*p.W1098X*) in *MYBPC3 *achieved a correction rate of approximately 3.65% of total mutations at 6 months post-treatment. Despite the modest editing efficiency, no pathological phenotype was observed during follow-up [74]. Further preclinical work using CRISPR-Cas9 systems with adenine base editors in mouse models carrying the *MYH7 c.1208G>A* mutation achieved correction of over 70% of mutant transcripts, with minimal off-target (bystander) editing. Serial echocardiographic assessments confirmed a reduction in myocardial hypertrophy [75, 76]. Notably, the *p.R403Q* variant alone accounts for about 0.5% of all sarcomeric mutations, representing an estimated 25,000 patients who may benefit from this therapeutic strategy [26].

Finally, gene silencing has been successfully applied to target *MYH6* mutations. In a heterozygous HCM mouse model carrying the *R403Q *mutation, administration of a mutation-specific siRNA (403i) resulted in an 80% reduction of the mutant transcript, while sparing approximately 80% of the wild-type allele. After 6 months of follow-up, treated mice showed no evidence of myocardial hypertrophy or fibrosis [77]. Importantly, this approach also demonstrated the ability to silence multiple mutations within the same gene by targeting shared single nucleotide polymorphisms, potentially broadening its applicability.

### 5.2 Future Prospects of Gene Therapy

While GT shows promising preclinical results in HCM, several challenges limit its clinical application.

Firstly, AAV vectors can trigger immune responses leading to hepatotoxicity, myocarditis, and neurotoxicity. Dosing must be carefully managed to avoid adverse effects, and treatment is contraindicated in patients with pre-existing neutralizing antibodies [78, 79]. Packaging capacity of AAV is limited to ~4.7 kb, necessitating complex multi-vector delivery for large transgenes or editing tools [76]. Alternative vectors are under investigation [80].

Secondly, CRISPR designs must be allele-specific to avoid off-target effects. Normal gene function must be preserved and oncogenesis evaded. The need for mutation-specific sgRNAs also raises concerns about the scalability and cost-effectiveness of therapies for rare variants. Polygenic or non-Mendelian HCM forms may not benefit.

Clinical trial design is complicated by small sample sizes, ethical concerns due to incomplete penetrance and variable expressivity, and uncertainties about timing and disease reversibility. Evaluation often requires invasive biopsies, with surrogate endpoints being necessary. Long-term efficacy, durability of single-dose treatments, and optimal therapeutic windows remain undefined. To date, the longest published preclinical follow up lasted just 34 weeks [71].

Finally, access and affordability present major obstacles. High development costs, regulatory complexity, and limited availability may restrict treatment to high-resource settings. Regulatory hurdles are sure to arise due to GT complexity, safety concerns and long-term effects. Specific regulatory frameworks must be created, with a likely long approval process.

Despite these significant barriers, GT still represents a therapeutic revolution, sparking the hope that, in the future, gene-targeted approaches may offer curative potential for select subtypes of HCM [10].

## 6. Conclusions 

During the last few years, a revolution in HCM has occurred; and a future revolution is to come. New SCD risk stratification methods incorporating technological advances and AI, are expected to emerge, improving ICD decision-making in HCM patients. As MI studies come to light, oHCM treatment algorithms may evolve, potentially positioning novel therapies as first-line options. Specific nHCM treatment is due to emerge. Septal ablation techniques will likely be reserved for non-responders, employing novel methods to improve outcomes and reduce complications. Finally, GT will soon become available for certain HCM genotypes enabling tailored, definitive treatments. Future prospects may even include preventive treatments for genetic carriers. HCM management will shift from phenotype-based to genotype-specific approaches, ushering us into the era of precision personalized medicine.

## Abbreviations

AAV, Adeno-Associated Viral Vectors; AI, Artificial Intelligence; ASA, Alcohol Septal Ablation; CMR, Cardiac Magnetic Resonance; CRISPR-Cas9, Clustered Regularly Interspaced Short Palindromic Repeats Associated Cas9 Nucleases; ECV, Extracellular Volume; EF, Ejection Fraction; GT, Gene Therapy; HCM, Hypertrophic Cardiomyopathy; ICD, Implantable Cardioverter-Defibrillators; KCCQ-CSS, Kansas City Cardiomyopathy Questionnaire–Clinical Summary Score; LAVI, Left Atrial Volume Index; LGE, Late Gadolinium Enhancement; LV, Left Ventricular; LVEF, Left Ventricular Ejection Fraction; LVMI, Left Ventricular Mass Index; LVOT, Left Ventricular Outflow Tract; LVOTO, Left Ventricular Outflow Tract Obstruction; MACE, Major Adverse Cardiovascular Event; MI, Myosin Inhibitors; mRNA, Messenger Ribonucleic Acid; nHCM, Non-Obstructive Hypertrophic Cardiomyopathy; oHCM, Obstructive Hypertrophic Cardiomyopathy; pVO_2_, Maximum Oxygen Consumption; PIMSRA, Percutaneous Intramyocardial Septal Radiofrequency Ablation; REMS, Risk Evaluation and Mitigation Strategy; SCD, Sudden Cardiac Death; sgRNA, single guide RNA; siRNA, small interfering RNA; SRT, Septal Reduction Therapy; TA-BSM, Transapical Beating-Heart Septal Myectomy; TTLA, Transthoracic Laser Ablation; VF, Ventricular Fibrillation; VT, Ventricular Tachycardia.
